# A Modified Brain MR Image Segmentation and Bias Field Estimation Model Based on Local and Global Information

**DOI:** 10.1155/2016/9871529

**Published:** 2016-08-29

**Authors:** Wang Cong, Jianhua Song, Kuan Luan, Hong Liang, Lei Wang, Xingcheng Ma, Jin Li

**Affiliations:** ^1^College of Automation, Harbin Engineering University, Harbin 150001, China; ^2^Electronic Engineering College, Heilongjiang University, Harbin 150080, China

## Abstract

Because of the poor radio frequency coil uniformity and gradient-driven eddy currents, there is much noise and intensity inhomogeneity (bias) in brain magnetic resonance (MR) image, and it severely affects the segmentation accuracy. Better segmentation results are difficult to achieve by traditional methods; therefore, in this paper, a modified brain MR image segmentation and bias field estimation model based on local and global information is proposed. We first construct local constraints including image neighborhood information in Gaussian kernel mapping space, and then the complete regularization is established by introducing nonlocal spatial information of MR image. The weighting between local and global information is automatically adjusted according to image local information. At the same time, bias field information is coupled with the model, and it makes the model reduce noise interference but also can effectively estimate the bias field information. Experimental results demonstrate that the proposed algorithm has strong robustness to noise and bias field is well corrected.

## 1. Introduction

As an important medical treatment technique, magnetic resonance imaging (MRI) has greatly enhanced the efficiency of the doctor's diagnosis and avoided the numerous anatomical surgeries or diagnostic laparotomies. In various applications of MR image, brain MR image has played a vital role in the detection and diagnosis of brain diseases. In practice, however, brain MR image has some deficiencies such as noise interference, intensity inhomogeneity, low contrast, and the partial volume effect of brain tissue. Therefore, it is very difficult to segment accurately brain MR images. Now, most of the image segmentation methods assume that the intensity distribution of MR images is uniform, only from the perspective of antinoise performance to segment MR image, which is bound to cause inaccurate segmentation result. For this reason, to achieve higher segmentation accuracy, it is necessary to estimate the bias field of MR images.

Currently, fuzzy *C*-means (FCM) clustering technique has been widely applied in image segmentation, and it has the advantages of unsupervised segmentation, simple arithmetic, and fast speed of computation [1–3]. Meanwhile, the algorithm also has shortcomings. It only considers single pixel information in the image while ignoring the influence of neighborhood spatial pixel, which is sensitive to noise. Therefore, many scholars put forward a series of improved algorithms [4–9]. Ahmed et al. [4] presented an algorithm that incorporated the neighboring spatial information of a pixel in local window into the objective function of the FCM algorithm to improve antinoise capability (called BCFCM). Cai et al. [5] modified BCFCM algorithm (called FGFCM) where the gray level value and local spatial information of adjacent pixels are all similarly considered in the measurement. Krinidis et al. [6] proposed a clustering algorithm called FLICM (fuzzy local information *C*-means), which introduces an adaptive control factor in its objective function without trial-and-error experiments, as well as obtaining better antinoise performance and image segmentation accuracy. However, the aforementioned algorithms only considered the local neighborhood information of the image without taking into account the global structural information of an image. As a result, the FCM algorithm with local spatial constraints generally cannot get satisfactory segmentation results.

In recent years, a few FCM algorithms with global constrained methods were presented [10–12]. Zhao et al. [11] proposed an efficient fuzzy clustering scheme, in which a nonlocal constraint item is introduced into the objective function of the improved FCM, and the global structure information of the image plays a very important role in the process of image segmentation. Feng et al. [12] proposed a nonlocal FCM clustering method with edge preservation, and the detail information of the image can be well preserved. The antinoise ability of these algorithms has been greatly improved for image segmentation. However, these algorithms ignored the correction of intensity nonuniformity in MR image.

In order to get rid of the influence of bias field, many studies have been launched in this respect by some scholars [13–20]. Wells et al. [13] suggested an adaptive estimation method of bias field using the expectation maximization model. The model can automatically segment each brain tissue, but it needs to accurately know the specific information of each tissue and results in high computational complexity. Sled et al. [14] proposed a nonparametric nonuniform intensity normalization (N3) approach in MR data. The method is iterative and seeks the smooth multiplicative field that maximizes the high frequency content of the distribution of tissue intensity, and it requires no a priori knowledge and can be applied to almost any MR image. Tustison et al. [15] improved N3 algorithm by replacing the B-spline smoothing strategy used in the original N3 framework with an advantageous alternative and modified the iterative optimization scheme to improve convergence performance (called N4ITK). N3 and N4ITK are available to the public, so they have been applied widely in medical image processing. However, N3 and N4ITK are only designed for bias correction, and the parameters estimated for B-spline are very time-consuming; they are generally used for image segmentation [16]. Li et al. [17] presented a scheme of bias field estimation and image segmentation based on coherent local intensity clustering (CLIC); the Gaussian kernel function is incorporated into the weighting of the local neighborhood in the model to measure the spatial distance between neighbor pixels in FCM objective function. Although CLIC model can correct the bias field, this algorithm only utilizes grayscale distribution information in local neighborhood without considering global information of the whole image. Therefore, CLIC algorithm is sensitive to noise and has lower segmentation accuracy.

Through the analysis mentioned above, it can be seen that FCM and its variants only use the difference between the central pixel and its neighboring pixels to calculate the local similarity measure. When both the central pixel and some of the neighboring pixels are abnormal pixels, it may fail to analyze the impact of each neighboring pixel on the local similarity measure exhaustively. In this condition, the local spatial information derived from the image may play a negative role in guiding the noisy image segmentation. However, the nonlocal information can take advantage of the high degree of redundancy in the image. In other words, every pixel in the image can find a set of samples with a similar neighborhood configuration to it. Then, the pixel under consideration could be influenced by the weighted averaging over these samples. If nonlocal adaptive spatial constraint term is introduced, then the objective function, the local neighborhood information, and global structure information of an image can be comprehensively utilized. Therefore, a novel model of simultaneous MRI brain tissue segmentation and bias field estimation is proposed in this paper, which includes two improvements. Firstly, an improved FCM objective function is constructed including local spatial constraint term and nonlocal constraint term, which can significantly enhance the antinoise performance and the segmentation accuracy. Secondly, the bias field was coupled with the model as a multiplicative additional field, and thus bias field can be effectively estimated from MR images to reduce the impact of intensity inhomogeneity for image segmentation.

## 2. Related Work

### 2.1. FCM Clustering with Spatial Constraints

In view of some drawbacks of standard FCM algorithm, a modified scheme is proposed by Ahmed et al. [4]. The objective function introduces a regularization term with spatial neighborhood constraints in the local window, and the new objective function is

(1) JFCM−S=∑i=1c ∑k=1Nuikmxk−vi2+αNΩ∑i=1c ∑k=1Nuikm∑r∈Ωkxr−vi2,

where *x*_*k*_ is the gray value of the *k*th pixel, *v*_*i*_ is the *i*th cluster center, *u*_*ik*_ is the fuzzy membership degree of the *k*th pixel belonging to the *i*th cluster and follows the constraint ∑_*i*=1_^*c*^ *u*_*ik*_ = 1, *N*_*Ω*_ is the number of neighborhood pixels, *x*_*r*_ is the gray value of the neighboring pixels in local window, and *Ω*_*k*_ represents the set of neighbors that exist in a window around *x*_*k*_. The parameter *m* is a weighting exponent controlling classification results, and *α* is a key parameter to control the balance between the first term and the second term in (1). By minimizing (1), *u*_*ik*_ and *v*_*i*_ can be derived as follows:

(2) uki=xk−vi2+α/NΩ∑r∈Ωkxr−vi2−1/m−1∑l=1cxk−vl2+α/NΩ∑r∈Ωkxr−vl2−1/m−1,vi=∑k=1Nuikmxk+α/NΩ∑r∈Ωkxr1+α∑k=1Nuikm.

### 2.2. FCM Clustering with Improved Fuzzy Partitions

FCM clustering with spatial constraints and its variants have better segmentation performance than the standard FCM algorithm. However, they still have some disadvantages. For example, their antinoise ability is not strong enough, and the execution efficiency of the algorithm is low, and the adaptive ability of related parameters is poor. Zhu et al. [10] proposed a more effective FCM clustering model (called GIFP_FCM), in which a novel energy function is built by introducing a new membership constraint item; the convergence speed and segmentation accuracy of GIFP_FCM algorithm get more significant enhancement than FCM. The corresponding objective function of GIFP_FCM is as follows:

(3) JGIFP_FCM=∑i=1c ∑k=1Nuikmxk−vi2+∑k=1Nak∑i=1cuikm1−uikm−1,

where *a*_*k*_ is an adjusted parameter for controlling the clustering performance of GIFP_FCM, *a*_*k*_ = *α*min⁡{‖*x*_*k*_ − *v*_*i*_‖^2^, *i* ∈ {1,2,…, *c*}}, and 0 < *α* < 1. By minimizing (3) using Lagrangian optimization, the fuzzy membership *u*_*ik*_ and clustering center *v*_*i*_ are derived as follows:

(4) uik=xk−vi2−ak−1/m−1∑l=1cxk−vl2−ak−1/m−1,vi=∑k=1Nuikmxk∑k=1Nuikm.

### 2.3. Bias Field Model

In practical application, affected by radio frequency coil, magnetic resonance equipment, and the variability of different brain tissue, intensity inhomogeneity generally appears in MR image. As a result, bias field correction is a very important task for MR image segmentation.

In MR image, bias field presents smooth variation of gray level of the pixels of the same tissue in spatial domain. Therefore, bias field can be regarded as a multiplicative component of an MR image. Let *X* be the observed image and *X*_0_ be the true image; *b* and *N* denote bias field and noise in the image, respectively. The model is as follows:

(5) $$\begin{array}{c} X=bX_{0}+N. \end{array}$$

### 2.4. CLIC Model

In CLIC model [17], a Gaussian kernel function is first used in local neighborhood by standard FCM algorithm to segment the MR image and then extended to the whole image. In the small neighborhood of an image, bias field can be approximated as constant; the pixel gray level can also be regarded as a constant. In this case, the bias field can be estimated when ignoring the additional noise in (5). The clustering criterion function of CLIC is

(6) $$\begin{array}{c} J_{CLIC}=\overset{c}{\underset{i=1}{\sum }}\;\overset{N}{\underset{k=1}{\sum }}u_{ik}^{m}\underset{r\in \Omega_{k}}{\sum }K\left(\;r-k\;\right){\left\|\;x_{k}-b\left(r\right)v_{i}\;\right\|}^{2}, \end{array}$$

where *b*(*r*) is the bias field, *K*(*r* − *k*) is the weight of a truncated Gaussian kernel assigned to the intensity *x*_*k*_, and the weighting function *K* is written as follows:

(7) $$\begin{array}{c} K\left(\;y\;\right)=\left\{\begin{array}{cc} \frac{1}{a}e^{-{\left|y\right|}^{2}/2\sigma^{2}}, & for\;\;\left|y\right|\leq \rho \\ 0, & else, \end{array}\right. \end{array}$$

where *σ* is the standard deviation of the Gaussian kernel, *ρ* is the radius of the adjacent window, and *a* is a constant to normalize the Gaussian kernel.

In CLIC model, image segmentation and bias field estimation can be completed simultaneously, but this model also has some drawbacks. First of all, as for the weighting of spatial measure, Gaussian kernel is only related to the local neighborhood of current target pixel, without taking into account the texture structure of the whole image. As a result, some of the pixels will get the error classification. Second, the model cannot effectively eliminate the influence of noise, because CLIC is modeled based on the standard FCM. Finally, a large number of nuclear convolution calculations result in high computational complexity in CLIC.

## 3. The Proposed Method

During MR brain image segmentation, the local neighborhood information and global structure information of the image should be used for ensuring the accuracy of the segmented results and robustness to noise. In this section, we reconstructed the objective function based on CLIC and GIFP_FCM algorithms, and a nonlocal regularization term is added in the modified model. In fact, the variation pattern of MR image pixels has certain regularity; that is, the brain tissue region has a strong global similarity, and it should be treated with nonlocal constraint.

### 3.1. Nonlocal Spatial Constraint

Given a noisy MR image, the similarity between the *k*th and *l*th pixels is measured by their gray value vectors *x*(*N*_*k*_) and *x*(*N*_*l*_), where *x*(*N*_*k*_) and *x*(*N*_*l*_) represent the square neighborhood of radius *s* and centered at pixels *x*_*k*_ and *x*_*l*_, respectively. The similarity of gray value vector is determined by Gaussian weighted Euclidean distance ‖*x*(*N*_*k*_) − *x*(*N*_*l*_)‖_2,*σ*_^2^; the expression is as follows:

(8) xNk−xNl2,σ2=∑p=12s+12σpxpNk−xpNl2,

where *σ* denotes the standard deviation of the Gaussian kernel, *x*(*N*_*k*_) denotes a window of radius *s* around *x*_*k*_, *x* ^(*p*)^(*N*_*k*_) is the *p*th element in this window, and *σ* ^(*p*)^ is defined as follows:

(9) $$\begin{array}{c} \sigma^{\left(p\right)}=\overset{s}{\underset{v=max\left(d,1\right)}{\sum }}\frac{1}{{\left(2v+1\right)}^{2}s}, \end{array}$$

where *y*_*p*_ = mod⁡(*p*, (2*s* + 1)) and *z*_*p*_ = floor(*p*, (2*s* + 1)) + 1. (*y*_*p*_, *z*_*p*_) denote the coordinates of the *p*th element in the square window *x*(*N*_*i*_), *d* = max⁡(|*y*_*p*_ − *s* − 1|, |*z*_*p*_ − *s* − 1|). Therefore, the similarity measure *S*_*kl*_ between the *k*th and *l*th voxels in the MR image is as follows:

(10) $$\begin{array}{c} S_{kl}=\frac{1}{Z_{k}}e^{\left(-{\left\|x\left(N_{k}\right)-x\left(N_{l}\right)\right\|}_{2,\sigma }^{2}/h\right)}, \end{array}$$

where *h* is a constant proportional to the noise deviation *σ*; it controls the decay of the similarity measure *S*_*kl*_ and satisfies 0 < *S*_*kl*_ < 1 and ∑_*l*∈*w*_*k*_^*u*^_ *S*_*kl*_ = 1;  *w*_*k*_^*u*^ represents a search window of radius *u* around the *k*th voxel. *Z*_*k*_ denotes a normalized constant; it is defined as follows:

(11) $$\begin{array}{c} Z_{k}=\underset{l\in w_{k}^{u}}{\sum }e^{\left(-{\left\|x\left(N_{k}\right)-x\left(N_{l}\right)\right\|}_{2,\sigma }^{2}/h\right)}. \end{array}$$

Therefore, for the *k*th pixel of the image, the nonlocal average method obtains its estimate value ${\overset{-}{x}}_{k}$ utilizing a weighted average method, and the expression is as follows:

(12) $$\begin{array}{c} {\overset{-}{x}}_{k}=\underset{l\in w_{k}^{u}}{\sum }S_{kl}x_{l}. \end{array}$$

### 3.2. Objective Function

The modified objective function incorporating local operation and nonlocal operation is as follows:

(13) Jm=∑i=1c ∑k=1Nuikm∑r∈ΩkKr−kxk−brvi2+∑i=1c ∑k=1Nakuikm1−uikm−1+βkuikm∑r∈ΩkKr−kx−k−brvi2,

where *u*_*ik*_ is the membership degree of fuzzy clustering, *v*_*i*_ is the *i*th cluster center, *Ω*_*k*_ represents a neighborhood window of radius *r* and centered at *x*_*k*_, and ${\overset{-}{x}}_{k}$ is the nonlocal calculated value of the *k*th pixel, and the definition of *K*(*r* − *k*) and *b*(*r*) is the same as (6). *β*_*k*_ is the weighting parameter to balance local coupling item and nonlocal regularization item of (13); the definition of parameter *β*_*k*_ is

(14) $$\begin{array}{c} \beta_{k}=\frac{\max\left(w_{k}^{u}\right)-\min\left(w_{k}^{u}\right)}{\max\left(w_{k}^{u}\right)}. \end{array}$$

**Theorem 1 .**

Assume ∑_*i*=1_^*c*^ *u*_*ik*_ = 1, 0 ≤ *u*_*ik*_ ≤ 1, and *m* > 1. By an optimization using Lagrange multiplier method in (13), *u*_*ik*_ and *v*_*i*_ can be derived as follows:

(15) uik=∑r∈ΩkKxk−bkvi2−ak+βkKx−k−bkvi2−1/m−1∑l=1c∑r∈ΩkKxk−bkvl2−ak+βkKx−k−bkvl2−1/m−1,

(16) vi=∑k=1N∑r∈ΩkKbkxk+βkx−kuikm∑k=1N∑r∈ΩkKbk21+βkuikm.

**Proof**

According to Lagrange multiplier method, (13) can be converted to unconstrained optimization problem:

(17) Luik,vi,bk,λk,ak,βk=∑i=1c ∑k=1Nuikm∑r∈ΩkKxk−bkvi2+∑i=1c ∑k=1Nakuikm1−uikm−1+βkuikm∑r∈ΩkKx−k−bkvi2+∑k=1Nλk1−∑i=1cuik.

Compute the partial derivative of *L* with respect to *u*_*ij*_ and *λ*_*k*_, respectively, and let ∂*L*/∂*u*_*ik*_ = 0 and ∂*L*/∂*λ*_*k*_ = 0; that is,

(18) ∂L∂uikmuikm−1∑r∈ΩkKxk−bkvi2+ak−makuikm−1+mβkuikm−1∑r∈ΩkKx−k−bkvi2−λk=0,

(19) ∂L∂λk1−∑i=1cuik=0.

From (18), we obtain

(20) uik=λk−akm∑r∈ΩkKxk−bkvi2−ak+βkKx−k−bkvi21/m−1.

By substituting (20) into (18), we obtain

(21) λk−akm−1/m−1=1∑l=1c∑r∈ΩkKxk−bkvl2−ak+βkKx−k−bkvl2−1/m−1.

By substituting (21) into (20), we obtain

(22) $$\begin{array}{c} u_{ik}=\frac{\sum_{r\in \Omega_{k}}^{}{\left(K{\left\|x_{k}-b_{k}v_{i}\right\|}^{2}-a_{k}+\beta_{k}K{\left\|{\overset{-}{x}}_{k}-b_{k}v_{i}\right\|}^{2}\right)}^{-1/\left(m-1\right)}}{\sum_{l=1}^{c}{\left(\sum_{r\in \Omega_{k}}^{}\left(K{\left\|x_{k}-b_{k}v_{l}\right\|}^{2}-a_{k}+\beta_{k}K{\left\|{\overset{-}{x}}_{k}-b_{k}v_{l}\right\|}^{2}\right)\right)}^{-1/\left(m-1\right)}}. \end{array}$$

Similarly, let ∂*L*/∂*v*_*i*_ = 0; that is,

(23) ∂L∂vi=∑k=1Nuikm·∑r∈ΩkKbkxk−bk2vi+βkbkx−k−bk2vi=0.

From (23), we obtain

(24) $$\begin{array}{c} v_{i}=\frac{\sum_{k=1}^{N}\sum_{r\in \Omega_{k}}^{}Kb_{k}\left(x_{k}+\beta_{k}{\overset{-}{x}}_{k}\right)u_{ik}^{m}}{\sum_{k=1}^{N}\sum_{r\in \Omega_{k}}^{}Kb_{k}^{2}\left(1+\beta_{k}\right)u_{ik}^{m}}. \end{array}$$

The theorem is completely proved.

### 3.3. Bias Field Estimation

To estimate the bias field *b*_*k*_ of the image in (17), we adopt the same analysis method and take the partial derivative of *L* with respect to *b*_*k*_. Let ∂*L*/∂*b*_*k*_ = 0; that is,

(25) ∂L∂bk=∑i=1cuikm·∑r∈ΩkKxkvi−vi2bk+βkx−kvi−vi2bk=0.

From (25), *b*_*k*_ can be obtained:

(26) $$\begin{array}{c} b_{k}=\frac{\sum_{i=1}^{c}\sum_{r\in \Omega_{k}}^{}Kv_{i}\left(x_{k}+\beta_{k}{\overset{-}{x}}_{k}\right)u_{ik}^{m}}{\sum_{i=1}^{c}\sum_{r\in \Omega_{k}}^{}Kv_{i}^{2}\left(1+\beta_{k}\right)u_{ik}^{m}}. \end{array}$$

### 3.4. The Algorithm Flow

The program flow of the proposed algorithm can be summarized as follows.

**Step 1 .**

The following are given: the number of clusters *c*, the exponent of fuzziness *m*, the radius of local window *s*, the radius of search window *u*, bias field *b*_0_ = 1, and stop criterion *ε*.

**Step 2 .**

Get the fuzzy cluster prototypes *V* ^(1)^ = {*v*_1_^(1)^, *v*_2_^(1)^,…, *v*_*c*_^(1)^} using *C*-means algorithm and set the iteration initial value *t* = 0.

**Step 3 .**

Compute nonlocal similarity measure *S*_*kl*_ using (10) and then obtain the nonlocal weighted average ${\overset{-}{x}}_{k}$ for the *k*th pixel using (12).

**Step 4 .**

Compute and update the membership degree *u*_*ik*_^(*t*)^ by (15).

**Step 5 .**

Compute and update the clustering prototypes *v*_*i*_^(*t*)^ by (15).

**Step 6 .**

Get the bias field estimate *b*_*k*_^(*t*)^ by (26).

**Step 7 .**

If max⁡‖*V* ^(*t*+1)^ − *V* ^(*t*)^‖ < *ε*, then output results; otherwise, go to Step 3 and set *t* = *t* + 1.

## 4. Experimental Results

To validate the validity of the proposed algorithm, several bias field estimation and image segmentation algorithms are taken as comparative methods. In all experiments, the parameter settings are as follows: the stop criterion *ε* = 0.001 and *m* = 2 and the radius of two windows *r* = 10 and *s* = 3, respectively.

### 4.1. Bias Field Correction

**Figure 1:**
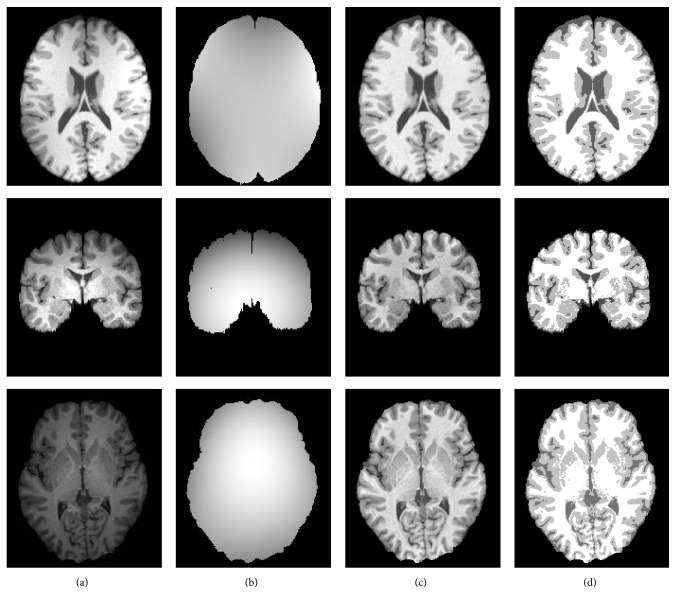
Bias field estimation and corresponding segmentation results. (a) Three MR images, (b) the estimated bias field, (c) the images of removed bias field, and (d) the segmented images.

First of all, the proposed method is applied to the 1.5T- and 3T-weighted brain MR images, and Figure 1 illustrates the experimental results. Column (a) shows three MR images with different level intensity nonuniformity (INU), column (b) shows the estimated bias field, column (c) shows the corrected images, and column (d) shows the segmentation results. In column (d), the brain tissue of corrected image becomes very homogeneous, and the cerebrospinal fluid (CSF), white matter (WM), and grey matter (GM) can be also clearly identified.

**Figure 2:**
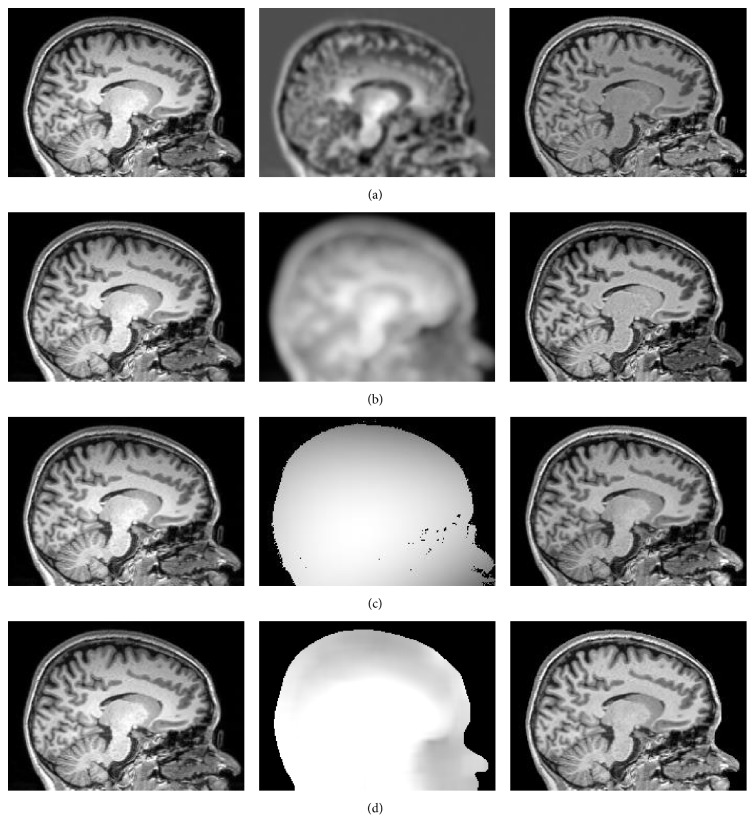
Comparison of the estimated bias fields of several algorithms. (a) BCFCM, (b) N3, (c) N4ITK, and (d) the proposed method.

**Figure 3:**
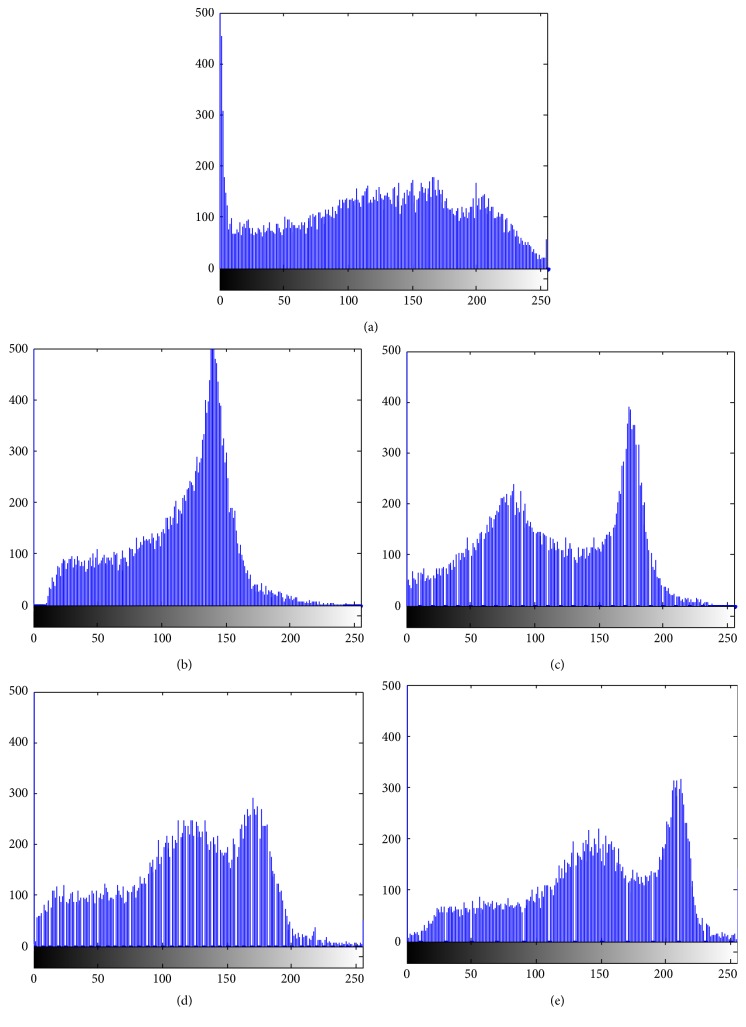
Histograms of original image and bias corrected images in Figure 2. (a) original image, (b) BCFCM, (c) N3, (d) N4ITK, and (e) our method.

In addition, Figure 2 compares results by BCFCM [4], N3 [14], N4ITK [15], and the proposed algorithm on MR images. The original images, estimated bias fields, and bias corrected images are shown in the first, second, and third columns of Figures 2(a)–2(d), respectively. The histogram of the original MR image and the histograms of the bias corrected images by BCFCM, N3, N4ITK, and our methods are shown in Figure 3. In the histograms, the right two significant peaks correspond to the GM and WM, respectively. The peak of the cerebrospinal fluid (CSF) is not distinct since its volume is relatively small. From the histograms of the bias corrected images recovered by BCFCM, N3, N4ITK, and our methods, we see that the histograms of specific tissues approximately satisfy Gaussian distribution but with significantly different variances. These results validate that our model is more consistent with the intensity distribution of the image with intensity inhomogeneity than other algorithms.

### 4.2. Antinoise Ability Analysis

**Figure 4:**
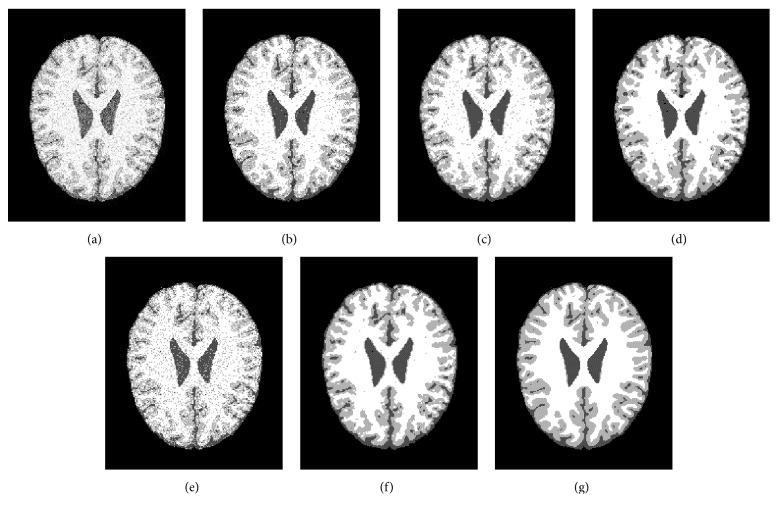
Antinoise ability comparison of the five algorithms. (a) Noisy image, (b) FCM, (c) BCFCM, (d) GIFP_FCM, (e) CLIC, (f) the proposed algorithm, and (g) ground truth.

In the second experiment, the images from BrainWeb simulated MR image database [21] are applied to analyze the antinoise ability of five algorithms (FCM, BCFCM [4], GIFP_FCM [10], CLIC [17], and our method). These simulated brain images are of T1-weighted contrast with a 1 mm slice thickness, 15% Rician noise, and no intensity inhomogeneities. The noisy MR images are segmented into four classifications: CSF, WM, GM, and the background. Before segmentation, the extracranial tissues were removed. The segmentation results using the five methods are shown in Figure 4, respectively.

Figure 4(a) is the MR image with 15% (*l* = 15) Rician noise. Figures 4(b)–4(f) show the corresponding experimental results by the five methods, respectively.  Figure 4(g) shows the ground truth. Figure 4 shows that the ability of our method is excellent at the preservation of image detail and robustness to noise, while the other four compared algorithms are relatively poor.

In order to quantitatively analyze the antinoise performance of the five algorithms, 8 MR images (the level of Rician noise ranges from 5% to 20%) are selected as the experimental samples. The statistical results (average values) of the Jaccard similarity (JS) [22] values of GM, WM, and CSF are shown in Table 1. JS was used for comparison and quantitative evaluation. Hence,

(27) $$\begin{array}{c} JS=\frac{\left|A_{i}\cap B_{i}\right|}{\left|A_{i}\cup B_{i}\right|}, \end{array}$$

where *A*_*i*_ denotes the set of pixels belonging to the *i*th class identified by the clustering algorithm, while *B*_*i*_ denotes the set of pixels belonging to the *i*th class in the ground truth. As a fuzzy similarity measure, the larger the JS value, the better the clustering performance. It can be seen from the experimental results in Table 1 that, with the increase of noise level of MR image, JS values of all algorithms are reduced. However, our method has higher values than the other four algorithms, and it illustrates that the proposed algorithm has better clustering performance and stronger robustness to noise.

### 4.3. Brain Tissue Segmentation of Noisy MR Image

**Figure 5:**
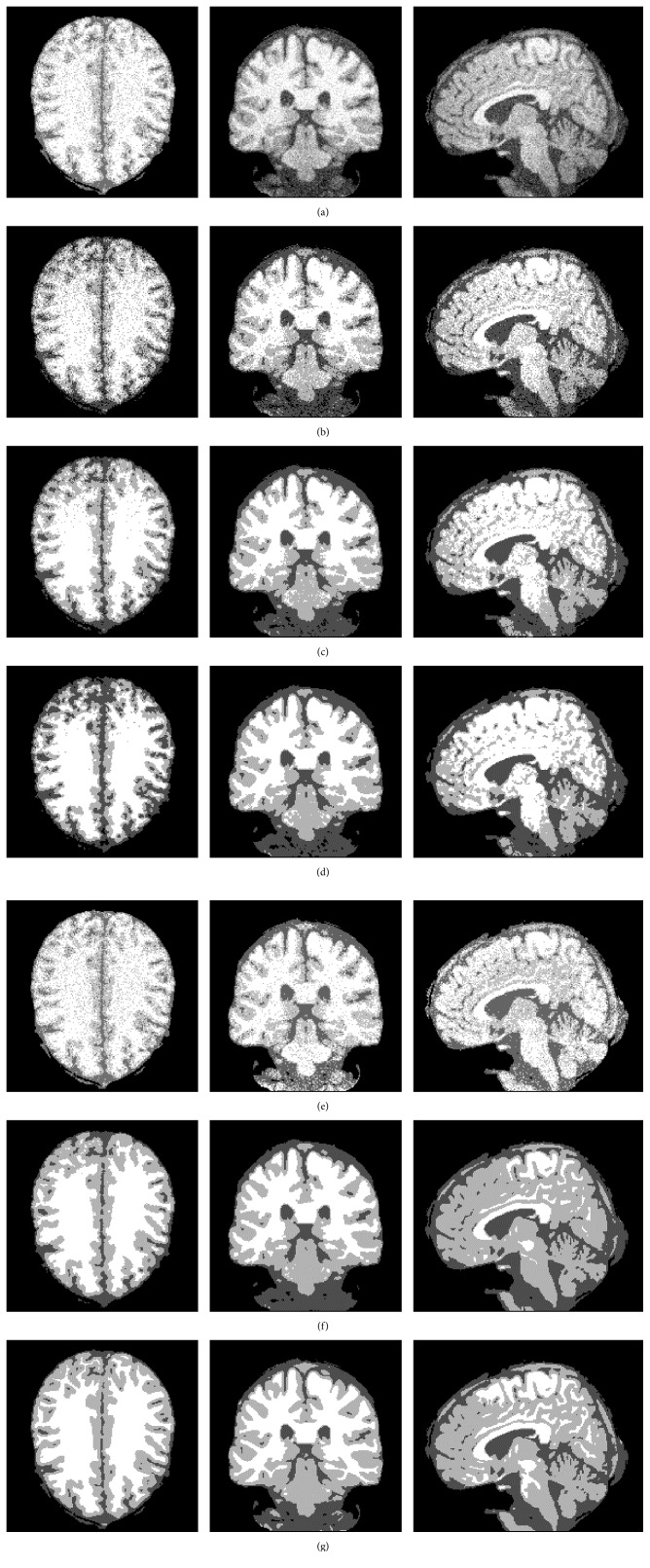
Experimental results for three MR images. (a) Noisy MR images, (b) FCM, (c) BCFCM, (d) GIFP_FCM, (e) CLIC, (f) the proposed method, and (g) ground truth.

In the third experiment, three MR images are selected for brain tissue segmentation by five algorithms, and these images are corrupted by 12% Rician noise and 40% intensity inhomogeneity. The noisy MR images are shown in Figure 5(a), and the corresponding segmentation results of FCM, BCFCM, GIFP_FCM, CLIC, and the proposed algorithm are shown in Figures 5(a)–5(f), respectively.  Figure 5(g) shows the ground truth of the three test images.  Figure 5 shows that the proposed algorithm not only can estimate bias field but also can effectively ensure segmentation accuracy of three MR images.

**Figure 6:**
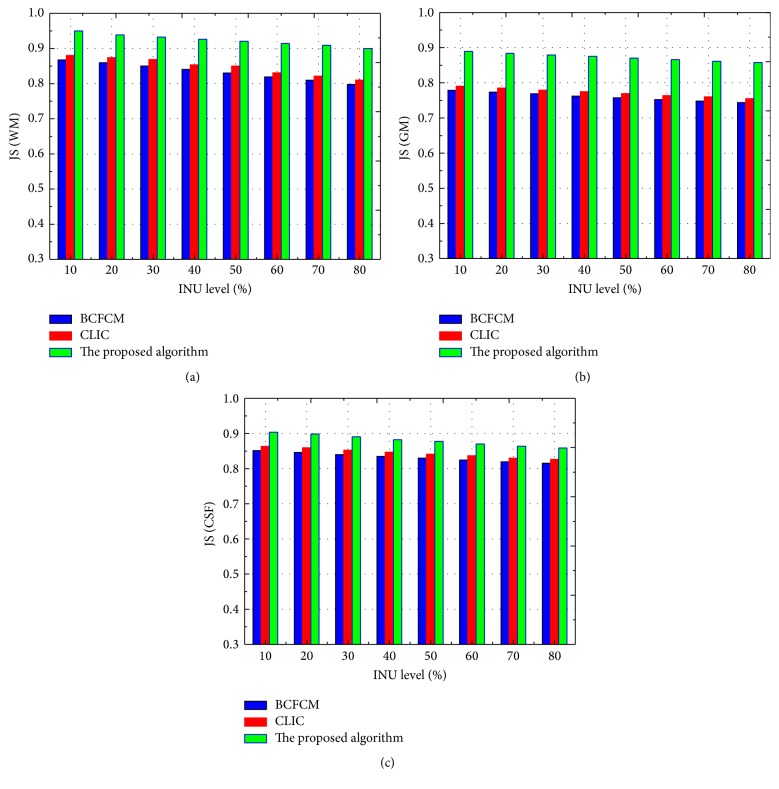
JS comparison of the brain tissue segmentation with three algorithms: (a) JS of WM segmentation, (b) JS of GM segmentation, and (c) JS of CSF segmentation.

To evaluate the segmentation accuracy with the variety of bias fields, we tested 10 MR brain images using three bias field estimation algorithms: BCFCM, CLIC, and the proposed method. The JS values of WM, GM, and CSF are compared when the INU level of bias fields varied from 10% to 80% for the MR images with noise 9%, and the comparison results are illustrated in Figure 6, respectively. It can be observed from Figure 6 by quantitative comparison that JS values of three algorithms are progressively smaller with the increase of the INU level, and our proposed model can preserve more image detail than the other two methods.

## 5. Conclusion

The traditional FCM algorithms with local information to suppress noise have some limitations in image segmentation. Due to the nonlocal weighted measure being unable to reflect the distance of real pixel points to the clustering center, a robust MRI brain tissue segmentation and bias field estimation model based on local and global information is proposed in this paper. During the process of calculating similarity measure, the algorithm utilized the nonlocal information to adjust parameters and reduce the difficulty of parameter setting; meanwhile, bias fields of MR brain image with intensity inhomogeneity is corrected. The segmentation results of the experiment showed that the proposed method presents stronger robustness to noise and accurately estimates the bias field, and more detailed structure of the MR brain image can be efficiently preserved.

## Figures and Tables

**Table 1:** Comparison of JS on simulated MR images with different levels of noise.

Algorithm	Tissues	5%	10%	15%	20%
FCM	WM	0.8373	0.7458	0.6403	0.5773
	GM	0.7189	0.5325	0.4211	0.3021
	CSF	0.8359	0.6751	0.5238	0.4001

BCFCM	WM	0.9022	0.8563	0.7459	0.6832
	GM	0.8754	0.7681	0.5765	0.4308
	CSF	0.8868	0.8354	0.6869	0.5369

GIFP_FCM	WM	0.9491	0.8993	0.8357	0.7365
	GM	0.9011	0.8377	0.7324	0.5793
	CSF	0.9268	0.8634	0.7957	0.7263

CLIC	WM	0.9007	0.8557	0.7461	0.6989
	GM	0.8766	0.7706	0.6013	0.5426
	CSF	0.8993	0.8457	0.6956	0.5536

The proposed algorithm	WM	**0.9513**	**0.9320**	**0.8697**	**0.7833**
	GM	**0.9295**	**0.8723**	**0.7838**	**0.6619**
	CSF	**0.9457**	**0.8803**	**0.8329**	**0.7937**
